# Supplementary material for: Host population structure and treatment frequency maintain balancing selection on drug resistance
Source: J R Soc Interface. 2017 Aug 23;14(133):20170295. doi: 10.1098/rsif.2017.0295 (PMC5582124; doi:10.1098/rsif.2017.0295)
Supplement: Electronic Supplementary Material [file rsif20170295supp1.pdf]

Supporting Information

Host population structure and treatment frequency maintain balancing selection on drug resistance

Sarah Cobey, Edward B. Baskerville, Caroline Colijn, William Hanage, Christophe Fraser, and Marc Lipsitch

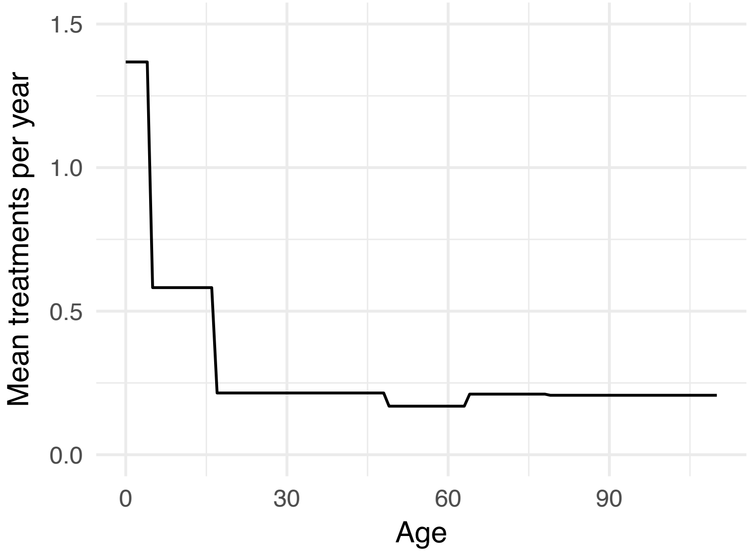

Figure S1: Mean number of treatments per year for each age group. The default treatment duration was 10 days (Table 1). When the treatment duration was varied, the total antibiotic use (number of treatment days $\times$ number of treatments per year) was kept constant for each age group, and the number of treatments per year was rescaled.

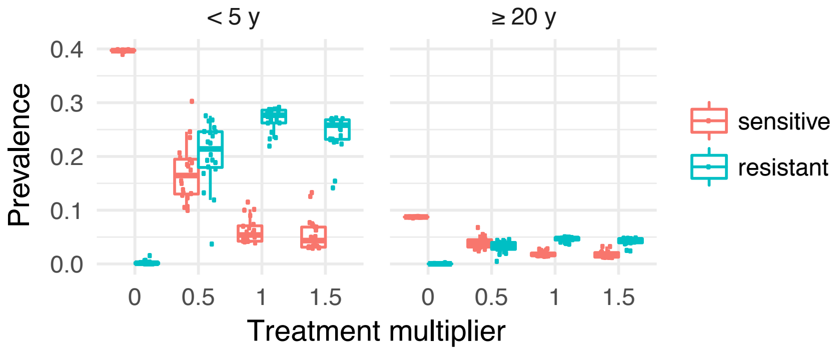

Figure S2: Prevalence of resistant and sensitive strains in young children (< 5 y) and adults (≥ 20 y) in the model with age-assortative mixing (AAM), age-specific treatment (AST), and pseudo-spatial immigration (PSI). The ratio of the duration of resistant to sensitive strain's durations in treated hosts (γ_r) was set to 4, and the fitness cost of resistance was 4% ($\xi = 0.96$) and acted on duration. Each point shows the mean fraction of each serotype that is resistant from one replicate simulation. Means were obtained by averaging the last 50 years.

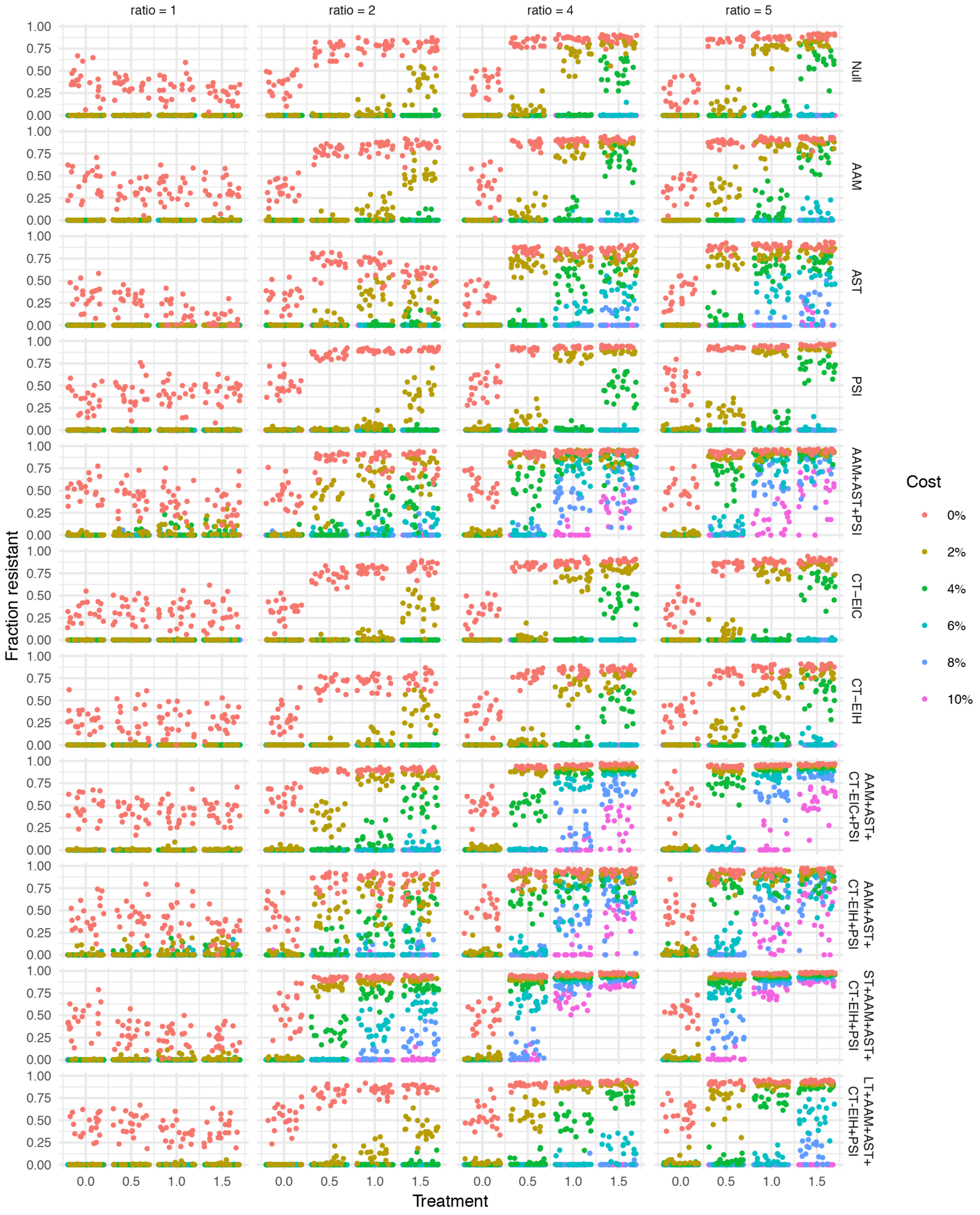

Figure S3: The fraction of resistant strains by treatment level and the ratio of resistant strains' duration compared to sensitive strains' in treated hosts. Each point corresponds to one simulation. AAM = age-assortative mixing, AST = age-specific treatment, PSI = pseudo-spatial immigration, CT = cotransmission, EIH = equally infectious hosts, EIC = equally infectious colonizations, trans = fitness cost in transmission instead of duration; ST = short treatment (high frequency), LT = long treatment (low frequency).

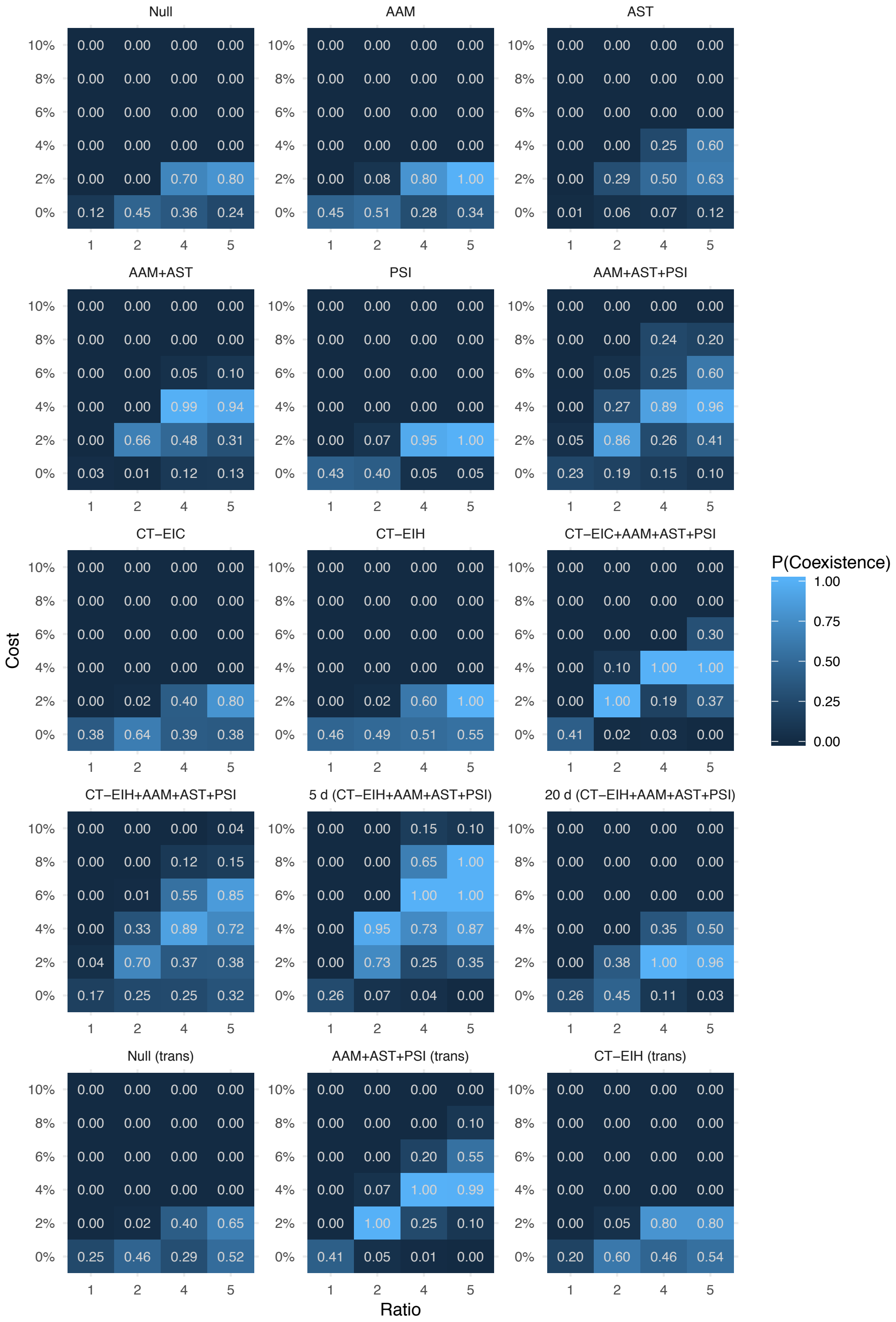

Figure S4: Fraction of replicate simulations producing coexistence for different fitness costs, ratios of the resistant to sensitive strain's duration in treated hosts, and models. Coexistence is defined as a resistant fraction of 2%-98% and a $\geq$ 10% increase in the fraction resistant over 50% to 150% of typical treatment rates (see Fig. S3). AAM = age-assortative mixing, AST = age-specific treatment, PSI = pseudo-spatial immigration, CT = cotransmission, EIH = equally infectious hosts, EIC = equally infectious colonizations, trans = fitness cost in transmission instead of duration.

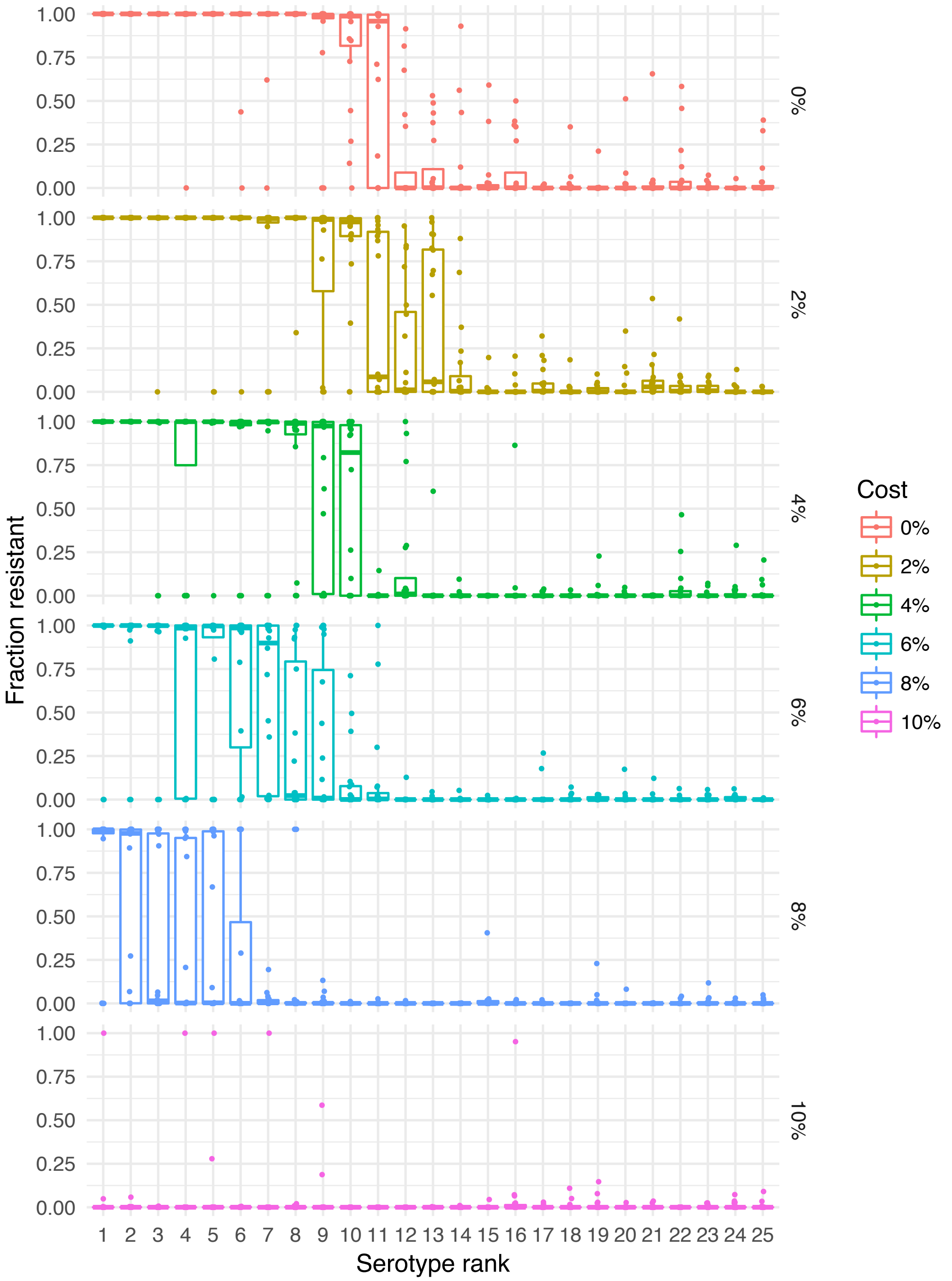

Figure S5: Resistance in adults ($\geq$ 20 y) by serotypes' fitness ranks for different costs of resistance in the model with age-assortative mixing (AAM), age-specific treatment (AST), pseudo-spatial immigration (PSI), and fitness cost in the duration of carriage. Treatment is held at 1.0, equivalent to U.S. rates. Each point shows the mean fraction of each serotype that is resistant from one replicate simulation. Means were obtained by averaging the last 50 years.

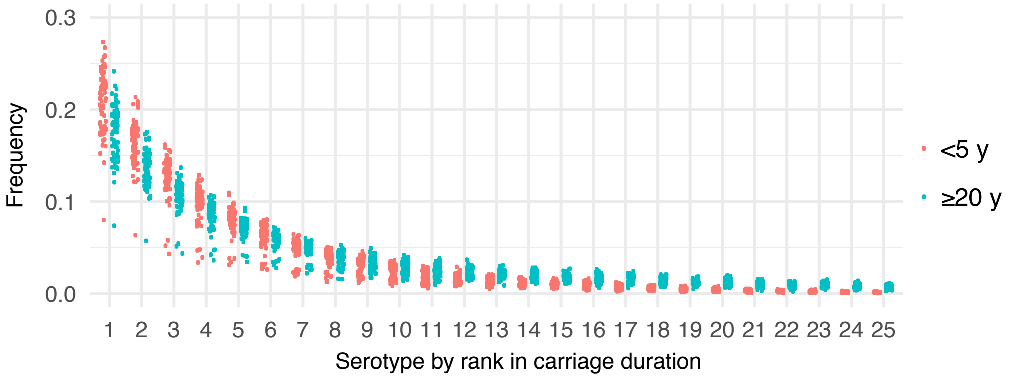

Figure S6: Frequencies of serotypes ranked by their carriage duration, from the longest-duration serotype (first) to the shortest-duration serotype (last), in children < 5 y and adults ≥ 20 y. Each point shows the mean frequency of each serotype (of all colonizations) from one replicate simulation of a model with age-assortative mixing (AAM) and age-structured treatment (AST) with treatment level 1 and a fitness cost of 2% ($\xi = 0.98$). Means were obtained by averaging frequencies from the last 50 years.

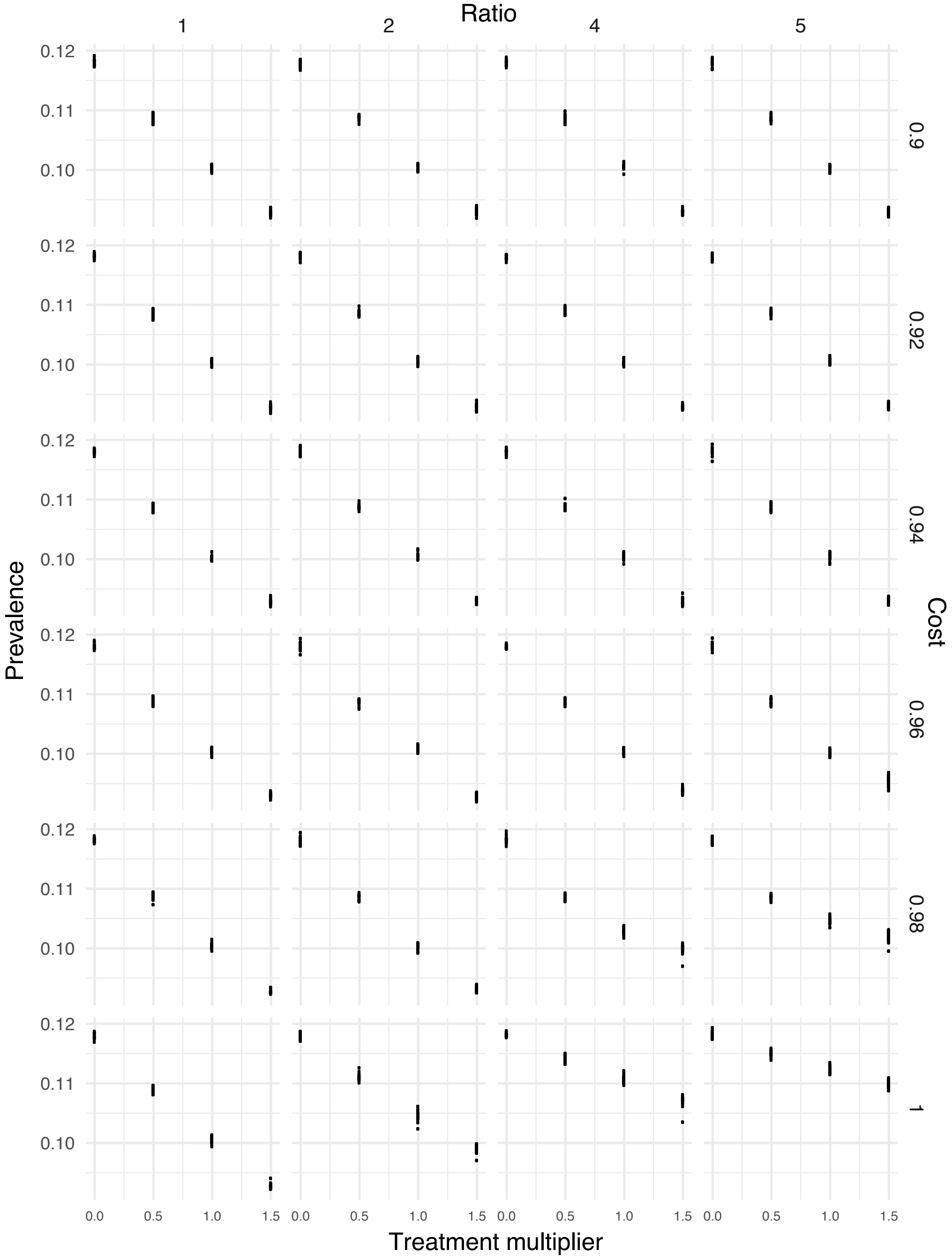

Figure S7: Mean carriage prevalence in the host population for the model with age-assortative mixing (AAM), age-specific treatment (AST), pseudo-spatial immigration (PSI), and the fitness cost of resistance in the duration of carriage. Each point shows the mean prevalence from one replicate simulation. Means were obtained by averaging the last 50 years.

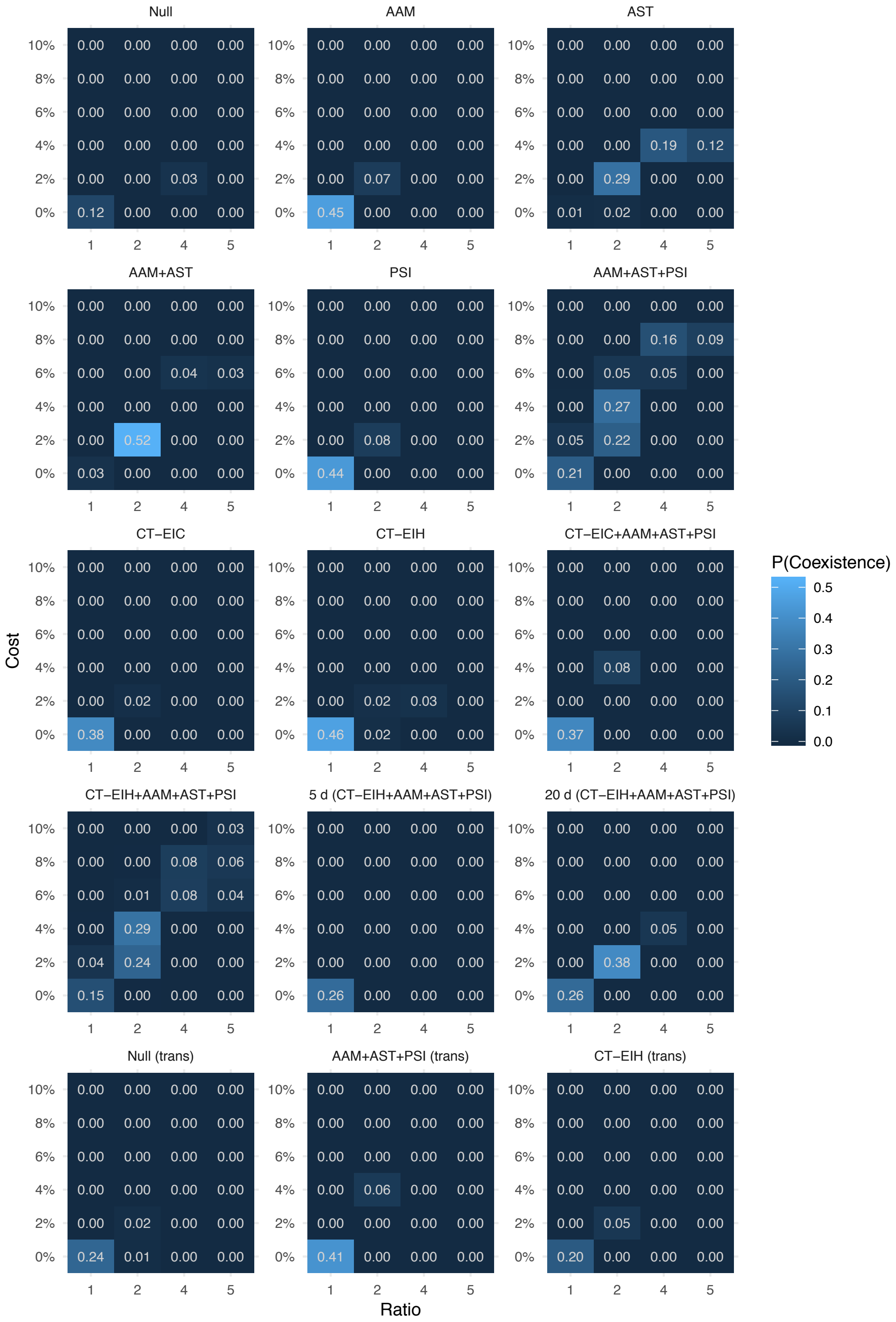

Figure S8: Fraction of replicate simulations producing coexistence for different fitness costs, ratios of the resistant to sensitive strain's duration in treated hosts, and models. Coexistence is defined as a resistant fraction of 2%-60% and a $\geq$ 10% increase in the fraction resistant over 50% to 150% of typical treatment rates (see Fig. S3). AAM = age-assortative mixing, AST = age-specific treatment, PSI = pseudo-spatial immigration, CT = cotransmission, EIH = equally infectious hosts, EIC = equally infectious colonizations, trans = fitness cost in transmission instead of duration.
